# Paroxysmal Nocturnal Hemoglobinuria as a Rare Cause of Chronic Asymptomatic Hemolysis: A Case Report

**DOI:** 10.7759/cureus.103221

**Published:** 2026-02-08

**Authors:** Patrícia Araújo, Iara Fazenda, Ana Frederica Parente, António Cardoso Fernandes, Carmélia Rodrigues

**Affiliations:** 1 Internal Medicine, Unidade Local de Saúde do Alto Minho, Viana do Castelo, PRT

**Keywords:** acquired mutations, anemia, clonal hematologic disorder, complement mediated hemolysis, paroxysmal nocturnal hemoglobinuria (pnh)

## Abstract

Paroxysmal nocturnal hemoglobinuria (PNH) is a rare acquired clonal hematologic disorder characterized by complement-mediated intravascular hemolysis, bone marrow failure, and an increased risk of thrombosis. Clinical presentation is heterogeneous, ranging from severe symptomatic disease to indolent and asymptomatic forms, which may delay diagnosis. We report the case of a 66-year-old asymptomatic male with long-standing hematologic abnormalities and an otherwise negative diagnostic workup, in whom PNH was diagnosed by flow cytometry. This case highlights the importance of a systematic approach to anemia and the need to consider rare etiologies when standard investigations are inconclusive.

## Introduction

Paroxysmal nocturnal hemoglobinuria (PNH) is a rare clonal disorder of hematopoietic stem cells caused by acquired mutations in the PIG-A gene [1]. This mutation leads to defective synthesis of glycosylphosphatidylinositol (GPI) anchors and subsequent deficiency of GPI-linked proteins, including CD55 and CD59, on blood cells [1,2]. The absence of these complement regulatory proteins renders erythrocytes susceptible to complement-mediated lysis.

Clinically, PNH is characterized by intravascular hemolysis, bone marrow failure, and a markedly increased risk of thrombosis, which remains the leading cause of mortality [2]. However, the disease spectrum is broad, and some patients may present with minimal symptoms or remain asymptomatic for prolonged periods, making diagnosis challenging [3].

Its occurrence is estimated to be 15.9 individuals per million worldwide, but some authors consider it to be underestimated as the disease remains undiagnosed in individuals with limited symptomatology. Typically, most patients fall in the age range of 30 years to 40 years [3].

We describe a case of PNH in a patient with chronic asymptomatic hematologic abnormalities, emphasizing the importance of maintaining diagnostic suspicion for rare causes of anemia and hemolysis, since PNH is frequently overlooked.

## Case presentation

A 66-year-old man with no significant past medical history, no regular medications, and no toxic habits was referred to an internal medicine outpatient clinic for evaluation of persistent laboratory abnormalities, including decreased hemoglobin of 11.2g/dL, increased mean corpuscular volume of 96.9 fL, and 81,000/μL platelets. Macrocytosis and thrombocytopenia had been documented since 2001 (Table 1). The patient was clinically asymptomatic, denying fatigue, dyspnea, chest pain, bleeding, bruising, or hemoglobinuria.

**Table 1:** Timeline of hematologic counts

Parameters	Nov 2001	Nov 2019	July 2020	Aug 2020	Normal Range
Hemoglobin (g/dL)	13.4	12.4	11.2	11.1	13.2 - 17.2
Mean Corpuscular Volume (fL)	104	101.2	96.9	99.1	80.1 – 96.1
Mean Corpuscular Hemoglobin (pg)	35.4	35.8	35	34.5	26.7 – 30.7
Red Cell Distribution Width (%)	17.1	14.3	14.7	14.6	<15
White blood cells (10^9^/L)	4560	4060	5540	3870	4000 – 10 000
Neutrophils (10^9^/L | %)	2800 | 61.5%	2600 | 63.6%	3400 | 61%	2400 | 61%	1500 – 8000 | 55 – 75%
Eosinophils (10^9^/L | %)	0 | 0.4%	0 | 0.5%	0 | 0.5%	100 | 2%	0 – 300 | 1 – 3.7%
Basophils (10^9^/L | %)	0 | 0.7%	0 | 0.2%	0 | 0.4%	0 | 1%	0 – 300 | 0 – 2%
Lymphocytes (10^9^/L | %)	1400 | 31.2%	1100 / 27.3%	1600 / 29.4%	1000 / 27%	800 – 4000 | 17 – 33%
Monocytes (10^9^/L | %)	300 | 6.3%	300 | 7.9%	500 | 8.3%	300 | 8%	0 – 1200 | 5 - 9%
Immature Granulocytes (10^9^/L | %)	-	0 | 0.5%	0 | 0.5%	0 | 1%	0 – 300 | 0 – 3%
Platelets (/μL)	96,600	76,000	81,000	71,000	150 000 – 400 000

The patient's family history was notable for pancytopenia in one of his brothers and father, although further data about this issue was not available; another brother died from sarcoidosis. Physical examination was unremarkable, with no jaundice, lymphadenopathy, hepatomegaly, or splenomegaly on palpation.

**Figure 1:**
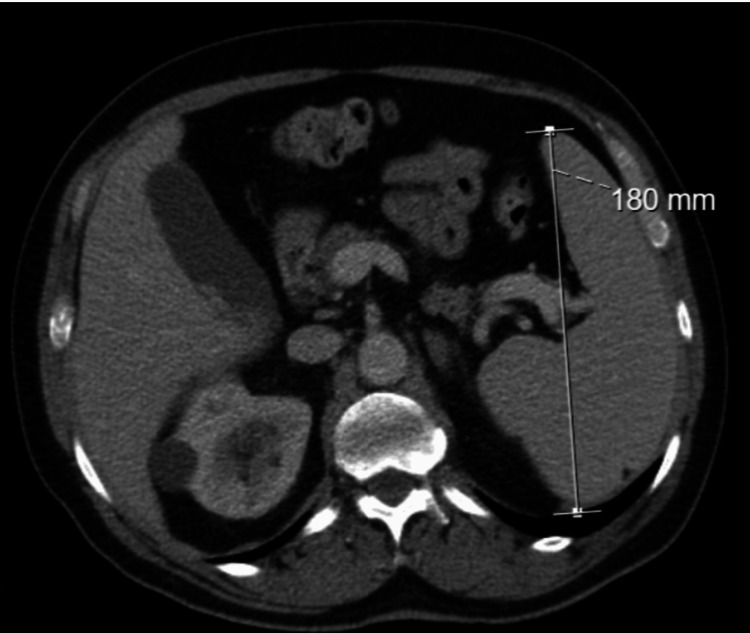
Abdominal computed tomography showing splenomegaly (~18 cm) without focal splenic lesions or associated lymphadenopathy.

Follow-up laboratory evaluation revealed mild pancytopenia with biochemical features suggestive of hemolysis (hemoglobin 11.1g/dL; mean corpuscular volume 99.1fL; white blood cells 3.870x10^9^/L; platelets 71000/μL; lactate dehydrogenase 594 U/L; total bilirubin 2.19mg/dL, direct bilirubin 0.7 mg/dL, reticulocytes 4.7%; haptoglobin <8mg/dL). Peripheral blood smear showed mild anisocytosis, rare tear-drop cells, macrocytic erythrocytes, slight anisochromy, and polychromasia without platelet aggregates. Immunoglobulin levels and serum protein electrophoresis were normal. Urinalysis showed no relevant abnormalities. Abdominal ultrasound and computed tomography revealed splenomegaly (18 cm) without focal lesions or lymphadenopathy (Figure 1).

A comprehensive workup excluded nutritional deficiencies, autoimmune hemolytic anemia, infectious diseases, thyroid dysfunction, liver disease, cardiac valvopathies, and hematologic or solid malignancy (Table 2).

**Table 2:** Laboratory findings The laboratory workup excluded nutritional deficiencies, autoimmune hemolytic anemia, infectious diseases, thyroid dysfunction, liver disease, cardiac valvopathies, and hematologic or solid malignancy

Parameters	Value	Normal Range
Urea (mg/dL)	47	17 – 43
Creatinine (mg/dL)	0.94	0.8 – 1.3
Sodium (mmol/L)	142	136 – 145
Potassium (mmol/L)	3.6	3.5 – 5.1
Total bilirubin (mg/dL)	2.19	0.3 – 1.2
Direct bilirubin (mg/dL)	0.7	< 0.5
Lactate dehydrogenase (U/L)	595	125 – 220
Alkaline phosphatase (U/L)	59	30 - 120
Gamma-glutamyl transferase (U/L)	22	< 38
Aspartate aminotransferase (U/L)	24	8 – 35
Alanine aminotransferase (U/L)	19	7 - 45
Albumin (g/dL)	4.7	3.2 – 4.6
Total proteins (g/dL)	6.9	6.4 – 8.2
Protein electrophoresis and immunofixation	No abnormalities	-
Immunoglobulin A (mg/dL)	300	60 - 400
Immunoglobulin G (mg/dL)	970	700 – 1600
Immunoglobulin M (mg/dL)	156	40 – 230
Haptoglobin (mg/dL)	< 8.0	14 - 258
Coombs test	Negative	Negative
Complement C3 (mg/dL)	100	90 - 180
Complement C4 (mg/dL)	33	12 – 40
Reticulocytes (%)	4.7	0.5 – 1.5
Erythrocyte sedimentation rate (mm)	21	2 – 8
Angiotensin-converting enzyme (U/L)	13.6	20 - 70
Iron (ug/dL)	107	50 – 170
Ferritin (ng/dL)	173.3	4.63 – 204
Total Iron-Binding Capacity (ug/dL)	280.7	250 – 425
Transferrin Saturation (%)	38	15 – 50
Folic acid (ng/dL)	18.4	3.1 – 20
Vitamin B12 (pg/dL)	616	187 - 883
Thyroid-Stimulating Hormone (U/mL)	1.52	0.35 – 4.94
Free T4 (U/mL)	0.83	0.7 – 1.48
Antinuclear Antibody	Negative	Negative
Antineutrophil Cytoplasmic Antibody	Negative	Negative
Human immunodeficiency viruses type 1-2	Non-reactive	Non-reactive
Hepatitis B surface antigen	Non-reactive	Non-reactive
Total Hepatitis C virus	Non-reactive	Non-reactive
Prothrombin time (seconds)	11.9	10.4 – 13.2
Activated Partial Thromboplastin Time (seconds)	26.3	25.3 – 31.6
International normalized ratio	1.06	-
Urine type II	No abnormalities	-

Given the unexplained hemolysis, flow cytometry was performed to assess for PNH. Immunophenotyping demonstrated PNH clones in neutrophils (99% CD16 weak/negative), monocytes (96% CD14 weak/negative), and erythrocytes (54% CD59 negative), confirming the diagnosis.

Anticoagulation therapy was initiated following referral to a specialized hematology clinic, with ongoing close surveillance. To date, he remains asymptomatic, without thrombotic events, and with stable pancytopenia.

## Discussion

Paroxysmal nocturnal hemoglobinuria is an uncommon disease [3]. Its clinical spectrum ranges from classical hemolytic PNH to subclinical forms associated with bone marrow failure syndromes, such as aplastic anemia (AA) or myelodysplastic syndromes (MDS) [2,4]. Intravascular hemolysis leads to nitric oxide depletion, resulting in smooth muscle dysfunction that manifests as abdominal pain, dysphagia, and erectile dysfunction [1].

Flow cytometry is the diagnostic gold standard, allowing precise quantification of glycosylphosphatidylinositol (GPI)-deficient cell populations across different hematopoietic lineages [3]. Current guidelines recommend periodic reassessment of clone size due to the potential for disease progression [2]. Bone marrow examination is not required for the diagnosis of PNH, but should be considered in patients with inappropriately low reticulocytosis, leukopenia, and/or thrombocytopenia in association with possible AA or MDS [5]. These two entities were considered based on the reported family history; however, they were inconsistent with the patient's clinical presentation.

An intriguing aspect of this case is the prolonged asymptomatic course despite a very large PNH clone and markedly elevated lactate dehydrogenase levels. PNH is characterized by significant clinical heterogeneity, and laboratory markers of hemolysis do not always correlate with symptom burden. Some patients exhibit an indolent phenotype, with chronic but compensated hemolysis due to increased bone marrow function, achieving minimal clinical manifestations for extended periods [1,6]. This case highlights that even patients with large PNH clones may remain asymptomatic for years. PNH should be considered in patients with unexplained cytopenias and laboratory evidence of hemolysis, even in the absence of hemoglobinuria or thrombotic events.

Splenomegaly is rare in classic PNH, which is characterized predominantly by complement-mediated intravascular hemolysis. Nevertheless, a secondary component of extravascular hemolysis, caused by C3 opsonization and reticuloendothelial clearance, occurs in untreated patients, although to a lesser extent than the primary intravascular phenotype [1]. Splenomegaly may stem from this mechanism as a result of prolonged evolution. Despite this, when splenomegaly is present, alternative explanations and PNH-related complications, such as splanchnic/portal venous thrombosis with portal hypertension, or overlapping marrow failure with secondary hypersplenism, must be considered.

Thrombosis is a major cause of morbidity and mortality in paroxysmal nocturnal hemoglobinuria and is strongly associated with intravascular hemolysis, elevated lactate dehydrogenase levels, and large PNH clone size. The role of primary prophylactic anticoagulation in patients without prior thrombotic events remains controversial [7]. Current literature and expert consensus emphasize that prophylactic anticoagulation should be individualized, particularly in patients with additional bleeding risk factors such as thrombocytopenia [8]. In this case, despite the absence of previous thrombosis, the presence of a very large granulocyte clone and persistent biochemical hemolysis suggested an increased thrombotic risk. Conversely, the platelet count of 71,000/μL raised concern for hemorrhagic complications. Following a multidisciplinary hematology review, anticoagulation was initiated after a careful risk-benefit assessment. This decision underscores the lack of universal guidelines for primary thrombosis prevention in PNH, and highlights the need for individualized management, especially in patients not yet meeting the criteria for complement inhibitor therapy.

The introduction of complement inhibitors, such as eculizumab and ravulizumab, has markedly improved survival and quality of life by reducing hemolysis and thrombotic risk [2]. However, these agents are generally reserved for patients with clinically significant hemolysis, thrombotic complications, or bone marrow failure, none of which were present in this case. Allogeneic hematopoietic stem cell transplantation remains the only curative treatment but is limited to selected patients due to its associated morbidity and mortality [4].

## Conclusions

This case highlights the diagnostic challenge posed by asymptomatic PNH and reinforces the importance of a systematic approach to anemia. PNH should be included in the differential diagnosis when standard testing is non-diagnostic. Early diagnosis and specialized hematology follow-up are essential to prevent potentially life-threatening complications, particularly thrombotic events, which may occur despite minimal symptoms.
